# A Positive Association between a Western Dietary Pattern and High LDL-C among Iranian Population

**DOI:** 10.34172/jrhs.2020.19

**Published:** 2020-07-25

**Authors:** Zahra Asadi, Meysam Moghbeli, Sayyed Saeid Khayyatzadeh, Maryam Mohammadi Bajgiran, Roshanak Ghaffarian Zirak, Reza Zare-Feyzabadi, Marziyeh Eidi, Mahdi Taheri bonakdar, Hafeze Davari, Ali Asghar Mahmoudi, Nazanin Sheikh Andalibi, Gordon A.Ferns, Hamideh Ghazizadeh, Majid Ghayour-Mobarhan

**Affiliations:** ^1^Department of Nutrition, Faculty of Medicine, Mashhad University of Medical Sciences, Mashhad, Iran; ^2^Student Research Committee, Mashhad University of Medical Sciences, Mashhad, Iran; ^3^Department of Medical Genetics and Molecular Medicine, School of Medicine, Mashhad University of Medical Sciences, Mashhad, Iran; ^4^Metabolic Syndrome Research Center, Mashhad University of Medical Sciences, Mashhad, Iran; ^5^Nutrition and Food Security Research Centre, Shahid Sadoughi University of Medical Sciences, Yazd, Iran; ^6^Brighton & Sussex Medical School, Division of Medical Education, Falmer, Brighton, UK; ^7^International UNESCO Center for Health-Related Basic Sciences and Human Nutrition, Mashhad University of Medical Sciences, Mashhad, Iran

**Keywords:** Dyslipidemia, Dietary patterns, FFQ

## Abstract

**Background:** The association between the presence of dyslipidemia and major dietary patterns was examined in an adult Iranian population.

**Study design:** A cross-sectional study.

**Methods:** This cross-sectional study was conducted among 4672 adults aged 35-65 yr old based on data from the Mashhad Stroke And Heart Atherosclerotic Disorder (MASHAD) Study initiated in 2010. Anthropometric and blood laboratory measurements were collected for all participants. Dietary intake was assessed using a validated 65-item food frequency questionnaire (FFQ). Dietary patterns were identified using factor analysis.

**Results:** The overall prevalence of dyslipidemia was 88% including elevated total cholesterol (38.9%), triglyceride (35.2%), low-density lipoprotein cholesterol (LDL-C) (35.3) or decreased level of high-density lipoprotein cholesterol (HDL-C) (68.9%). After adjusting for potential confounding factors, participants with higher scores for a Western pattern with lower physical activity level and educational attainment, and higher current smoking habit, increased the risk of having a raised LDL-C (OR=1.17; 95% CI: 1.02, 1.34; *P* =0.02). However, there was no significant association between adherence to this dietary pattern and other types of dyslipidemia. There was no significant association between a balanced dietary pattern and dyslipidemia and its components (OR=0.90; 95% CI: 0.68, 1.18; *P* =0.431).

**Conclusion:** Dyslipidemia was more prevalent among individuals with higher consumption of a western dietary pattern. A direct association was found between adherence to Western dietary pattern and LDL-C level.

## Introduction

Elevation of serum lipid concentrations [total cholesterol, triglycerides (TG), low-density lipoprotein cholesterol (LDL-C), or a reduction of high-density lipoprotein cholesterol (HDL-C)] is defined as dyslipidemia^1^. The prevalence of high total cholesterol (total cholesterol ≥ 200 mg/dl) varies with regions so that this disease is more widespread in Europe (54%) and the United states (48%) compared to the Southeast Asia (29%)^2^. In a systematic meta-analysis study, the prevalence of dyslipidemia defined as high total cholesterol (≥ 200 mg/dl), high TG (≥ 150 mg/dl), high LDL-C (≥ 130 mg/dl) or a low HDL-C (< 40 in males and < 50 in females) among Iranian adults were reported 41.6%, 46%, 35.5% and 43.9% respectively^3^. Compared with other Asian countries, dyslipidemia has a high prevalence in Iran. Dyslipidemia contributes to atherosclerotic cardiovascular disease (CVD)^4^. A positive association was found between serum total cholesterol level and myocardial infarction (MI) risk among Iranian males^5^. CVD is associated with a high mortality and morbidity rate in Iran and is a major public health problem^6^. The management of dyslipidemia requires changing lifestyle behaviors such as weight reduction, increasing physical activity and modifying dietary intake ^7^.

The associations of dietary patterns with diseases have drawn the attention of scientists recently since dietary patterns are representative of the overall diet of individuals and may be used to indicate changes in dietary intake over time ^8^.

An association was reported between a ‘prudent’ dietary pattern and serum HDL-C in both sexes, whereas the intake of coffee, fat and sweets was associated with a high LDL-C in men, while no association was found between westernized pattern with several dyslipidemia components including high LDL-C or low HDL-C ^9^. A study among preschool children demonstrated an increased prevalence of high LDL-C in those with lower scores of a mixed pattern similar to ‘balanced’ dietary pattern with consumption of all food groups ^10^. In contrast, in a cross-sectional study of 9725 Korean adults, a ‘balanced’ dietary pattern was not significantly associated with dyslipidemia ^11^, and in a sample of 583 Iranian female teachers, a ‘western’ dietary pattern was not found to be associated with dyslipidemia, whilst a healthy dietary pattern was associated with a decreased the risk of dyslipidemia ^12^.

Several studies have considered the dietary sources of energy and their association with abnormal lipid profile. A prospective study of 18 countries reported an association of higher dietary total fat with increased most of lipid profile components, and higher dietary carbohydrate intake with decreased most of these components ^13^. In a recent study, a positive association was found between a high percentage of energy from carbohydrate and high TG and low HDL-C and a negative association with higher total cholesterol and LDL-C among Korean adults. However, dietary fat intake increased the risk of high total cholesterol and high LDL-C and decreased the risk of low HDL-C and high TG ^14^.

We aimed to identify major dietary patterns among an Iranian population and the relationship between these dietary patterns with various types of dyslipidemia.

## Methods

### Study population

This cross-sectional study was based on data from the Mashhad Stroke And Heart Atherosclerotic Disorder (MASHAD) Study initiated in 2010 to explore the association of cardiovascular disease (CVD) with different risk factors among 9704 individuals of Mashhad City with the age range of 35 to 65 years ^15^. Subjects with incomplete FFQ, energy consumption of <800 kcal or >4200 kcal, and missing data on blood lipid profile (TC, TG, LDL-C and HDL-C) were excluded from the study. In addition, subjects taking medication to lower serum lipids were excluded. The remaining 4672 subjects were included, which was about 48% of total population of MASHAD Study.

### Dyslipidemia diagnosis

According to the American Association of Clinical Endocrinologists' guidelines, dyslipidemia has been defined as a serum total cholesterol ≥200 mg/dl or TG ≥150 mg/dl or LDL-C ≥130 mg/dl or low HDL-C <40 mg/ dL in males and <50 mg/ dL in females ^16^.

### Anthropometric measurements

Weight (kg), height (cm), waist circumference (cm), hip circumference and mid-upper arm circumference were precisely measured by health care professionals according to standard protocols^17^. Waist-to-hip ratio (WHR) was calculated by dividing WC to HC. Body mass index (BMI) was calculated by dividing weight to height square.

### Laboratory evaluation

The blood samples of all participants were collected after 14 h overnight fast. Fasting blood glucose (FBG), TC, TG, LDL-C and HDL-C were assessed by enzyme-based methods on an automated analyzer ^1^. A standard mercury sphygmomanometer was used to measure blood pressure. The blood pressure was assessed twice with a 30-min interval and the average of the two measurements was decided to be the final blood pressure.

### Dietary pattern assessment

Dietary intake of all subjects was recorded by a 65-item validated food frequency questionnaire (FFQ)^18^. The questionnaire was comprised of 65 food items with the frequency of consumption (per day, week, month, rarely and never) and serving size for each food item. The questionnaire of all subjects was completed by expert nutritionists through a face-to-face interview. The analysis was performed based on principal components factor analysis (PCA) method^19^. Varimax rotation was used to keep factors uncorrelated^20^. To decide on the number of components of dietary patterns, factors with an eigenvalue >1, the interpretability and the screen test were used^20^. Factor scores were measured by weighting the food groups/ items according to their factor loadings^20^. Foods/food groups with factor loadings <0.20 were excluded. Finally, two major dietary patterns were identified, labeled as Balanced and Western dietary patterns. Every subject received a score for each dietary pattern. Thereafter, the dietary patterns were used to divide the group into tertiles (T1, T2, and T3) for data comparisons. The first tertile (T1) was defined as the reference group.

### Physical activity level

The physical activity level of participants was measured by completing The James and Schofield human energy requirements equations. In this questionnaire, the questions were separated into work time activities, leisure time activities and resting time in bed ^21^.

### Other variables

Information about socio-demographic (gender, age, marital status and education), medical history (medication treatment) and lifestyle (smoking status) variables were collected through a comprehensive questionnaire at baseline.

### Statistical analysis

All statistical analysis were performed using SPSS ver. 16.0 (SPSS, Chicago, IL). The chi-square, analysis of variance (ANOVA), and Kruskal-Wallis tests with a Bonferroni Post-Hoc correction were used to report distribution differences for qualitative and quantitative data respectively across tertiles of the dietary patterns. Sex-adjusted means for quantitative variables across each dietary pattern tertile were calculated using univariate model. Odds ratios (ORs) and 95% confidence intervals (CIs) for dyslipidemia were calculated using multinomial logistic regression across tertiles of dietary patterns, taking the lowest tertile group as the reference group. The calculated ORs were adjusted for potential confounders including sex, age, body mass index (m/kg^2^), physical activity level, smoking status (never, past, and current), education level, marital status and total energy intake. A *P* -value <0.05 was considered as significant.

## Ethical approval

This study was approved by the Human Research Ethics Committee of Mashhad University of Medical Sciences (No. 85134).

## Results

The data was analyzed for 4672 men and women of MASHAD study population. Major characteristics of participants according to demographic and lifestyle variables, physical measurements and lipid and metabolic markers are presented in Table 1. Mean BMI was 27.94 ± 4.67 kg/m^2^ and the prevalence of hypertension and diabetes was 12% and 9.6% respectively. The prevalence of dyslipidemia was high, is estimated to be approximately 88% in total (a high serum TC, high TG, high LDL-C and low HDL-C were observed among 38.9%, 35.2%, 35.3% and 68.9% of the study population respectively).

**Table 1:** Characteristics of the participants

**Demographic and lifestyle**	**Number**	**Percent**
Gender, male	1969	42.1
Age range (yr)		
35-44	1493	32.0
45-54	1933	41.4
55-65	1245	26.6
Marriage status		
Single/ divorced/ widow	335	7.2
Married	4337	92.8
Education level		
Low	2551	54.8
Moderate	1770	38
High	336	7.2
Smoking status		
Nonsmoker	3195	68.4
Ex-smoker	477	10.2
Current smoker	1000	21.4
Physical activity level		
Sedentary	1131	25.3
Low active	1213	27.2
Active	1645	36.8
Very active	478	10.7
Dyslipidemia	4103	87.8
**Physical measurements**	**Mean**	**SD**
Body mass index (kg/m²)	27.9	4.6
Waist circumference (cm)	95.2	12.1
Hip circumference (cm)	103.5	9.1
Waist-to-hip ratio	0.9	0.0
Mid-upper arm circumference (cm)	30.3	3.4
Lipid and metabolic markers		
Fasting blood glucose (mg/dl)	92.5	38.6
Serum Cholesterol (mg/dl)	192.4	38.5
Serum low density lipoprotein cholesterol (mg/dl)	119.3	34.5
Serum high density lipoprotein cholesterol (mg/dl)	41.8	9.3
**laboratory measurements**	**Median**	**Interquartile range**
Serum triglyceride (mg/dl)	122	87 to 176

Two major dietary patterns were identified among the study population: A ‘‘Balanced’’ dietary pattern was characterized by high positive loadings for the consumption of green leafy vegetables, other vegetables, fruits, dairy products, red meats, poultry, legumes, nuts and seafoods. The second pattern labeled ‘‘Western’’ dietary pattern showed positive loadings for the consumption of sugar, egg, fast foods, tea, snacks, potato, carbonated beverages, pickled foods, organ meat, nuts and butter (Table 2).

**Table 2:** Factor-loading matrix for the major factors (dietary patterns) identified by using food consumption data from the food-frequency questionnaire used in the MASHAD cohort study

**Food groups**	**Balanced pattern**
Other vegetables	0.621
Green vegetables	0.589
Fruits	0.543
Dairy	0.484
Red meats	0.429
Poultry	0.336
Legumes	0.285
Nuts	0.271
Sea foods	0.261
**Food groups**	**Western pattern**
Sugars	0.552
Eggs	0.479
Fast foods	0.460
Tea	0.415
Snacks	0.410
Potato	0.394
Carbonated beverages	0.368
Pickled foods	0.337
Organs meat	0.309
Butter	0.281
Refined grains	-
Whole grains	-
Coffee	-

Foods or food groups with factor loadings <0.20 for both factors were excluded

In the Balanced dietary pattern, there were significant between-group differences concerning education level, smoking status, weight, height, BMI, waist circumference (WC), waist-to-hip ratio (WHR) and triglyceride (TG) concentration (Table 3). Comparing the highest versus the lowest tertile of the Balanced dietary pattern, the participants tended to have higher educational attainment (*P* <0.001), be current smokers (*P* <0.001) whereas, they had a lower WC (*P* =0.001) and WHR (*P* =0.001).

**Table 3:** Baseline characteristics and nutrient intake of participants according to tertiles of balanced dietary pattern

**Categorical variables**	**T1**		**T2**		**T3**		***P*** **value**			
	**Number**	**Percent**	**Number**	**Percent**	**Number**	**Percent**	**Total**	**T1 vs T2**	**T1 vs T3**	**T2 vs T3**
Gender							0.005	0.005	0.005	0.005
Female	1058	55.6	1113	55.9	471	60.6				
Male	845	44.4	878	44.1	307	39.4				
Education							0.001	0.001	0.001	0.001
Low	870	46.1	1411	70.9	270	34.7				
Moderate	747	39.6	480	24.1	336	43.2				
High	271	14.3	100	5.0	172	22.1				
Smoking status							0.001	0.001	0.001	0.001
Non smoker	1337	70.2	1296	65.1	562	72.2				
Ex – smoker	207	10.9	204	10.2	66	8.5				
Current smoker	359	18.9	491	24.7	150	19.3				
Physical activity level							0.028	0.028	0.028	0.028
Sedentary	491	26.9	434	22.7	206	28.1				
Low active	491	26.9	530	27.8	192	26.2				
Active	660	36.2	721	37.7	264	36.0				
Very active	181	10.0	225	11.8	72	9.7				
Marriage status							0.003	0.003	0.003	0.003
Single/divorced/widow	43	5.4	120	6.3	172	8.6				
Married	747	94.6	1771	93.7	1819	91.4				
Dyslipidemia							0.410	0.410	0.410	0.410
Total (N)	1686	88.6	1737	87.2	687	88.3				
**Continuous variables**	**Mean**	**SD**	**Mean**	**SD**	**Mean**	**SD**	**Total**	**T1 vs T2**	**T1 vs T3**	**T2 vs T3**
Age, year ^a^	49.29	0.18	48.98	0.18	49.57	0.28	0.171	0.676	1.000	0.224
Body mass index (kg/ m²)^a^	28.23	0.10	27.56	0.10	28.23	0.16	0.001	0.001	1.000	0.001
Cholesterol (mg/dl)^a^	193.04	0.88	191.35	0.86	193.60	1.37	0.247	0.506	1.000	0.492
LDL (mg/dl)^a^	118.61	0.79	120.51	0.77	117.89	1.23	0.104	0.258	1.000	0.214
HDL (mg/dl) ^a^	41.97	0.21	41.86	0.20	41.80	0.32	0.876	1.000	1.000	1.000
Energy (kcal/day)	2061.42	12.03	1781.46	11.76	2458.18	18.82	0.001	0.001	0.001	0.001
Carbohydrate (g/day) ^b^	273.37	39.30	296.49	36.01	251.53	51.98	0.001	0.001	0.001	0.001
Protein (g/day) ^b^	75.20	10.33	69.70	8.71	80.29	15.64	0.001	0.001	0.001	0.001
Fat (g/d)^b^	67.14	14.86	60.27	12.97	73.36	18.83	0.001	0.001	0.001	0.001
Cholesterol (mg/day)^b^	212.80	69.72	192.86	63.96	233.16	95.84	0.001	0.001	0.001	0.001
Fiber (g/day)^b^	26.33	7.35	25.33	7.35	27.43	8.78	0.001	0.001	0.001	0.001
**Continuous variables**	**Median**	**Interquartile** **range**	**Median**	**Interquartile** **range**	**Median**	**Interquartile** **range**	**Total**	**T1 vs T2**	**T1 vs T3**	**T2 vs T3**
Triglyceride (mg/dl)	124	89 to 179	118	85 to 169	128	89 to 184	0.001	0.001	1.000	0.001

T1: first tertile; T2: second tertile; T3: third tertile of the balanced dietary pattern. LDL: Low density lipoprotein cholesterol; HDL: density lipoprotein cholesterol

The nutrient intake of individuals was compared across tertiles for the balanced dietary pattern (Table 3). Individuals in the highest tertile of the Balanced pattern consumed higher energy, protein, total fat, cholesterol and fiber and lower carbohydrate (*P* <0.001).

Between-group comparison of the tertiles for the Western dietary pattern showed that there were significant differences among participants considering age, education level, smoking status, physical activity level (PAL), height, mid-upper arm circumference (MAC), prevalence of dyslipidemia, TG and low-density lipoprotein cholesterol (LDL-C) (Table 4). Those in the second tertile for the Western pattern were younger and participants of the highest tertile were less likely to have high educational attainment compared to the lowest tertile (*P* <0.001). Current smoking and sedentary lifestyle were more prevalent among those with a high western pattern score (*P* <0.001). Comparing anthropometric measurements, those in the upper tertile of Western pattern have higher height (*P* <0.001) and MAC (*P* =0.010). As we expected, the prevalence of dyslipidemia was higher in the last tertile of Western pattern (*P* <0.001), despite lower TG of the highest tertile compared to the first tertile (*P* =0.002).

**Table 4:** Baseline characteristics and nutrient intake of participants according to tertiles of Western dietary pattern

**Categorical variables**	**T1**		**T2**		**T3**		***P *** **value**			
	**Number**	**Percent**	**Number**	**Percent**	**Number**	**Percent**	**Total**	**T1 vs T2**	**T1 vs T3**	**T2 vs T3**
Gender							0.001	0.001	0.001	0.001
Female	1267	66.2	247	37.2	1191	56.9				
Male	647	33.8	417	62.8	903	43.1				
Education							0.001	0.001	0.001	0.001
Low	1077	56.4	305	46.2	1170	56.1				
Moderate	684	35.8	320	48.4	764	36.6				
High	149	7.8	36	5.4	152	7.3				
Smoking status							0.001	0.001	0.001	0.001
Non smoker	1443	75.4	358	53.9	1394	66.6				
Ex – smoker	186	9.7	88	13.3	203	9.7				
Current smoker	285	14.9	218	32.8	497	23.7				
Physical activity level							0.001	0.001	0.001	0.001
Sedentary	393	21.6	228	35.5	482	25.5				
Low active	462	25.4	200	31.2	520	27.5				
Active	743	40.7	156	24.3	704	37.2				
Very active	224	12.3	58	9.0	185	9.8				
Marriage status							0.001	0.001	0.001	0.001
Single/divorced/widow	170	8.9	27	4.0	138	6.6				
Married	1737	91.1	645	96.0	1955	93.4				
Dyslipidemia							0.001	0.001	0.001	0.001
Total	1724	41.9	545	13.3	1841	44.8				
**Continuous variables**	**Mean**	**SD**	**Mean**	**SD**	**Mean**	**SD**	**Total**	**T1 vs T2**	**T1 vs T3**	**T2 vs T3**
Age (yr) ^a^	51.35	0.18	46.90	0.30	47.97	0.17	0.001	0.001	0.001	0.001
Body mass index (kg/ m²)^a^	28.14	010	27.76	0.18	27.82	0.10	0.046	0.199	0.076	1.000
Cholesterol (mg/dl)^a^	193.62	0.88	189.30	1.50	192.30	0.83	0.043	0.042	0.826	0.244
LDL (mg/dl)^a^	119.03	0.79	115.99	1.35	120.60	0.75	0.010	0.164	0.445	0.009
HDL (mg/dl) ^a^	41.61	0.21	41.98	0.35	42.12	0.20	0.197	1.000	0.223	1.000
Energy (kcal/day)	1724.13	11.25	2631.32	19.10	2077.22	10.76	0.001	0.001	0.001	0.001
Carbohydrate (g/day) ^b^	274.49	37.02	282.57	60.67	283.14	42.23		0.001	0.001	0.001
Protein (g/day) ^b^	77.20	10.19	68.85	15.71	72.11	9.97		0.001	0.001	0.001
Fat (g/d)^b^	65.27	12.92	66.75	21.98	64.78	15.40	0.018	0.008	0.579	0.012
Cholesterol (mg/day)^b^	194.53	56.57	244.88	110.95	207.90	68.99		0.001	0.001	0.001
Fiber (g/day)^b^	27.17	6.96	23.48	8.73	25.94	7.80		0.001	0.001	0.001
**Continuous variables**	**Median**	**Interquartile range**	**Median**	**Interquartile range**	**Median**	**Interquartile range**	**Total**	**T1 vs T2**	**T1 vs T3**	**T2 vs T3**
Triglyceride (mg/dl)	122	87 to 176	118	79 to 175	120	87 to 169	0.002	0.008	0.002	0.575

Abbreviations: T1: first tertile, T2: second tertile, T3: third tertile of Western dietary pattern.

Participants with a high Western pattern diet, had higher dietary energy, carbohydrate and cholesterol while they consumed less fiber and protein compared with those with lower Western pattern scores (*P* <0.001).

The balanced dietary pattern showed no significant association with age-adjusted and multivariable-adjusted ORs (95% CI) of dyslipidemia or its components (Table 5). Although crude and age-adjusted ORs for a high serum TG were decreased in the second tertile of the balanced pattern compared to the reference tertile, after adjusting for all possible confounders this significant association was disappeared.

**Table 5:** Odds ratio (95% confidence intervals) of dyslipidemia according to tertiles of balanced dietary pattern

**Variables**	**Number**	**Crude OR (95% CI)**	***P*** **value**	**Adjusted OR (95% CI)** ^a^	***P*** **value**
Dyslipidemia					
First tertile	1686	1.00		1.00	
Second tertile	1737	0.88 (0.73, 1.07)	0.960	0.95 (0.77, 1.17)	0.732
Third tertile	687	0.97 (0.75, 1.26)	0.829	0.90 (0.68, 1.18)	0.431
High cholesterol					
First tertile	746	1.00		1.00	
Second tertile	748	0.93 (0.82, 1.06)	0.272	0.98 (0.85, 1.12)	0.308
Third tertile	323	1.10 (0.93, 1.30)	0.267	1.08 (0.90, 1.29)	0.383
High triglyceride					
First tertile	712	1.00		1.00	
Second tertile	657	0.82 (0.72, 0.94)	0.015	0.86 (0.75, 1)	0.056
Third tertile	302	1.05 (0.88, 1.25)	0.574	1.04 (0.86, 1.25)	0.688
High low density lipoprotein cholesterol					
First tertile	652	1.00		1.00	
Second tertile	721	1.09 (0.95, 1.24)	0.315	1.14 (0.99, 1.31)	0.366
Third tertile	285	1.11 (0.93, 1.32)	0.243	1.11 (0.92, 1.33)	0.266
Low high density lipoprotein cholesterol					
First tertile	1110	1.00		1.00	
Second tertile	1135	0.95 (0.83, 1.07)	0.726	1.09 (0.92, 1.29)	0.515
Third tertile	453	0.99 (0.84, 1.18)	0.949	1.01 (0.81, 1.26)	0.911

^a^ ORs were adjusted for age, sex, body mass index, physical activity level, smoking status, education level, marital status, and total energy intake.

After adjustment for age and other possible confounding factors, ORs of dyslipidemia were decreased in the second tertile of Western pattern versus the first tetile, however, these associations were not significant in the third tertile of Western pattern compared to the first tertile. We also found lower crude and age-adjusted ORs of high TC, but not multivariable adjusted OR, in T2 versus T1 of Western pattern.

Although crude and age-adjusted ORs of high serum TG were significantly decreased across tertiles of Western pattern (ORs = 0.78; 95% CI: 0.69, 0.89; *P* <0.001), however after adjustment for possible confounding factors this association was no longer significant. The age-adjusted ORs of a high serum TG in the highest versus lowest tertile of the Western pattern was 0.83 (95% CI: 0.73, 0.94; *P* =0.005, and the multivariable ORs of high serum TG was 0.81 (95% CI: 0.71, 1.01; *P* =0.062).

We also found positive association between adherence to Western pattern and LDL-C. The multivariable ORs of high LDL-C in the highest versus lowest tertile of Western dietary pattern was 1.17 (95% CI: 1.02, 1.34; *P* = 0.020).

The Western pattern was negatively correlated with HDL-C in the present study. The crude ORs of low HDL-C in the second tertile of Western pattern was 2.27 (95% CI: 1.87, 2.75; *P* <0.001) compared to the lowest tertile. Whereas, the age-adjusted ORs of low HDL-C in the second versus first tertile of Western dietary pattern was 2.22 (95% CI: 1.82, 2.69; *P* <0.001). Moreover, the crude ORs of low HDL-C in the highest tertile of Western pattern was 1.20 (95% CI: 1.05m 1.36; *P* =0.005) compared to the lowest tertile. Whereas, the age-adjusted ORs of low HDL-C in the highest versus lowest tertile of Western dietary pattern was 1.17 (95% CI: 1.03, 1.33; *P* =0.014). However, there was no significant association between a Western pattern and multivariable ORs of low HDL-C (OR= 0.86 (95% CI: 0.73, 1.01) *P* =0.067) (Table 6).

**Table 6:** Odds ratio (95% CIs) of dyslipidemia according to tertiles of Western dietary pattern

**Variables**	**Number**	**Crude OR (95% CI)**	***P*** **value**	**Adjusted OR (95% CI)** ^a^	***P*** **value**
Dyslipidemia					
First tertile	1724	1.00		1.00	
Second tertile	545	0.50 (0.39, 0.65)	0.001	0.64 (0.49, 0.84)	0.001
Third tertile	1841	0.80 (0.66, 0.98)	0.030	0.86 (0.70, 1.07)	0.185
High cholesterol					
First tertile	794	1.00		1.00	
Second tertile	223	0.71 (0.59, 0.86)	0.001	0.98 (0.80, 1.19)	0.572
Third tertile	800	0.87 (0.77, 1.00)	0.060	1.05 (0.91, 1.20)	0.493
High triglyceride					
First tertile	745	1.00		1.00	
Second tertile	230	0.83 (0.69,1.00)	0.084	0.84 (0.69, 1.03)	0.054
Third tertile	696	0.78 (0.69, 0.89)	0.001	0.81 (0.71, 1.01)	0.062
High low density lipoprotein cholesterol					
First tertile	686	1.00		1.00	
Second tertile	204	0.79 (0.65, 0.95)	0.013	0.96 (0.78, 1.18)	0.834
Third tertile	768	1.04 (0.91, 1.18)	0.583	1.17 (1.02, 1.34)	0.020
Low high density lipoprotein cholesterol					
First tertile	1016	1.00		1.00	
Second tertile	478	2.27 (1.87, 2.75)	0.001	0.98 (0.76, 1.26)	0.098
Third tertile	1204	1.20 (1.05, 1.36)	0.005	0.86 (0.73, 1.01)	0.067

^a^ ORs were adjusted for age, sex, body mass index, physical activity level, smoking status, education level, marital status, and total energy intake.

## Discussion

In the present study, the two major dietary patterns were distinct among the study population and were a combination of healthy and unhealthy food items. This could have contributed to the lack of significant associations of these dietary patterns with dyslipidemia. The balanced dietary pattern that showed positive loading factors for vegetables, fruits, dairy products, poultry, legumes, nuts and seafoods as well as red meats, was not related to abnormal lipid profile. The Western pattern showed positive loading factors for sugars, fast foods, tea, snacks, potato, carbonated beverages, pickled foods, organ meat, butter, nuts and egg increased the risk of high LDL-C.

In the current study, we observed a significant difference of education levels between the highest and lowest tertiles of the balanced dietary pattern, in which the group in the highest tertile of this dietary pattern had noticeably higher levels of university education compared to the lowest tertile (22.1% vs 14.3%). However, another study reported that there was inverse correlation between college education levels and healthy diet, in which the females who have college educations tend toward fast foods and unhealthy diets ^22^. Moreover, those participants who were in the highest tertile of Western dietary pattern had a higher education level than those in the lowest tertile ^23^.

We also observed a significant difference in smoking habit between the highest and lowest tertiles (23.7% vs 14.9%) of western dietary pattern. There were complex relationships between the presence of dyslipidemia and daily energy and fat intakes in the balanced and western dietary patterns. Although consumption of dietary total energy and fat were higher in the last tertile of the balanced dietary pattern compared to the first tertile, adherence to this healthy dietary pattern was correlated with lower prevalence of dyslipidemia in our results. On the other hand, adherence to western pattern with a lower dietary intake of total fat was associated with a higher dyslipidemia prevalence. These results are consistent with previous studies reported that high dietary fat intake is not an unavoidable factor for dyslipidemia^24,25^.

The psychological/ behavioral factors may explain these findings; the cases with dyslipidemia may have a greater tendency to decrease their daily energy and fat intakes compared to the cases who have normal lipid profiles. It may be expected to see higher physical activity among individuals with dyslipidemia compared to the other cases as we have observed among our cases. Lack of awareness and education about dyslipidemia can be a barrier to a healthy lifestyle. The educated cases with high awareness of their health status had more tendency for healthy diet and behavior ^26^. Indeed such behaviors can also be associated with the levels of education among the cases, in which the majority of highly educated cases were observed in the first tertile of the balanced dietary pattern compared to the other tetiles. Behavioral factors can affect the levels of serum cholesterol and susceptibility to specific diseases^27,28^. The type A behavior pattern (TABP) is associated with serum cholesterol levels and LDL-C ^29^. Type D behavior pattern is also characterized by negative emotions such as depression, psychological stress, and anxiety ^28^. Type D personality is associated with CVD and metabolic syndrome^30,31^, related to the plasma lipid levels ^31^. Dyslipidemia is also associated with type D personality through the cortisol as a mediator between type D personality and hyperlipidemia ^32^. Psychological stress can also increase the cardiovascular risk through unhealthy behaviors including smoking and a sedentary lifestyle or overactivation of autonomous sympathetic nervous system^33,34^. Occupational stress is significantly associated with dyslipidemia ^35^. We observed that although the highest tertile of the balanced dietary pattern had higher daily energy, fats, and cholesterol intake than the lowest tertile, such cases had lower incidences of dyslipidemia.

There are some other important factors involving in dyslipidemia such as daily fiber intake which was higher among those with the highest score of Balanced pattern. This highlights the inevitable role of fiber as a treatment option for dyslipidemia. Fiber is derived from plants that are generally resistant toward the human digestive enzymes. Dietary fiber intake reduces risk of different diseases such as hypertension, diabetes, and obesity^36-38^. Moreover, it improves serum lipid concentrations ^39^ and weight loss ^40^. High-dose fiber intake has beneficial influence on cholesterol cardiovascular risk factors^41^. Additionally, a significant relationship was shown between dietary Trans fatty acid and total sugar intake with adiposity^42^.

The balanced pattern, as the healthy pattern, was not correlated to dyslipidemia while the western pattern was positively associated with high LDL-C risk among a subpopulation of Iranian adults.

We observed some inconsistencies between the energy intake and daily fat intake and the prevalence of dyslipidemia in subjects with balanced dietary patterns associated with the daily fiber intake. Moreover, psychological factors such as job stress and awareness can also be involved in such inconsistencies between the balanced dietary pattern and dyslipidemia which require further psychological assessments. Factor analysis, as an exploratory method, was used to obtain major dietary patterns of the study population and factor interpretation was made based on subjective decision that affects the interpretation of the results.

There were some limitations in the present study: the design of the study was cross-sectional and thus demonstrated uncertain causality between dietary patterns and dyslipidemia.

## Conclusion

A balanced dietary pattern with high intake of green leafy vegetables, other vegetables, fruits, dairy products, red meats, poultry, legumes, nuts, and seafoods showed no significant association with dyslipidemia and its components, while adherence to Western dietary pattern with high intake of sugar, egg, fast foods, tea, snacks, potato, carbonated beverages, pickled foods, organs meat, and butter was positively associated with LDL-C level among the study population. More studies are required to confirm these findings.

## Acknowledgements

We would like to thank the Mashhad University of Medical Science Research Council for its financial supports. The study was approved by the Ethics Committee of Mashhad University of Medical Sciences (Ethics number: IR.MUMS.REC.1386.250).

## Conflict of interest

The authors have no conflict of interest to disclose

## Funding

This work was supported by a grant (Majid Ghayour Mobarhan) from Mashhad University of Medical Science (MUMS), Iran.

## Highlights

- The overall prevalence of dyslipidemia was reported 88% in Iranian population.
- Dyslipidemia was more prevalent among individuals with higher consumption of a western dietary pattern.
- In participants with higher scores for a western pattern increased the risk of having a raised LDL-C.
- There was no association between a balanced dietary pattern with high intake of dietary fiber and dyslipidemia.
